# C-Reactive Protein Level Predicts Cardiovascular Risk in Chinese Young Female Population

**DOI:** 10.1155/2021/6538079

**Published:** 2021-12-01

**Authors:** Ruifang Liu, Fangxing Xu, Qian Ma, Yujie Zhou, Tongku Liu

**Affiliations:** ^1^Department of Cardiology, 12th Ward, Beijing Anzhen Hospital, Capital Medical University, Beijing Institute of Heart Lung and Blood Vessel Disease, Beijing Key Laboratory of Precision Medicine of Coronary Atherosclerotic Disease, Clinical Center for Coronary Heart Disease, Capital Medical University, Beijing 100029, China; ^2^The Center of Cardiology, Affiliated Hospital of Beihua University, Jilin, Jilin 132011, China

## Abstract

**Background:**

C-reactive protein (CRP) is one of the most common oxidative indexes affected by many diseases. In recent years, there have been many studies on CRP, but the relationship between CRP levels and the cardiovascular risk in the Chinese young female population is still unclear. The purpose of this work is to explore the predictive value of CRP for the cardiovascular risk in the Chinese young female population.

**Methods:**

The study is conducted by 1 : 1 case-control to retrospectively analyze 420 young women with acute coronary syndrome (ACS group) who underwent percutaneous coronary intervention (PCI) and 420 young women (control group) who underwent coronary angiography (CAG) to exclude coronary heart disease from January 2007 to December 2016. All patients are divided into three subgroups according to CRP values: subgroup 1: CRP < 1.0 mg/L (*n* = 402); subgroup 2: 1.0 mg/L ≤ CRP ≤ 3.0 mg/L (*n* = 303); subgroup 3: CRP > 3.0 mg/L (*n* = 135). The levels of CRP were observed in the two groups and three subgroups.

**Results:**

A total of 840 patients were analyzed. The mean duration of follow-up was 66.37 ± 30.06 months. The results showed that the level of CRP in the ACS group was significantly higher than that in the control group (1.30 ± 1.70 vs. 3.33 ± 5.92, respectively, *p* < 0.001), and patients with higher CRP levels were associated with a significantly increased rate of major adverse cardiovascular events (MACE) (7.0% vs. 8.9% vs. 19.30%, respectively, *p* < 0.05). After adjustment for baseline covariates, CRP level was still an independent predictor for the incidence of MACE, either as a continuous variable or as a categorical variable. There was a significantly higher rate of all-cause mortality and myocardial infarction in patients with higher CRP values during follow-up.

**Conclusions:**

The research results show that high CRP is associated with increased risk of ACS in the Chinese young female population. Risk stratification with CRP as an adjunct to predict clinical risk factors might be useful in the Chinese young female population.

## 1. Introduction

Coronary heart disease (CHD) has become one of the top leading causes of death among Chinese adults [1]. The cardiovascular (especially CHD) morbidity and mortality have been rising in women from 1990 to 2020 [2–4]. Acute coronary syndrome (ACS) is a serious event for CHD. Among men and women with ACS, there are important dissimilarities in clinical presentation, cardiovascular risk factors and receiving medical care and unfavorable outcomes after percutaneous coronary intervention (PCI) is performed [5, 6]. Compared with men, women with ACS are receiving too little attention at present and have a higher risk of death and rate of major adverse cardiac events (MACE) following PCI [7, 8]. On the pathogenesis of ACS, previous studies have suggested that various inflammatory-related factors including oxidative stress may lead to vascular endothelial damage, which plays an important role in the process of atherosclerosis and makes important contributors to the development of ACS [9, 10]. As a marker of systemic inflammation, C-reactive protein (CRP) has been proven to be associated with increased relative risks of cardiovascular events in ACS patients [11]. However, thus far, no data are available regarding the relationship between CRP levels and the incidence of ACS in the Chinese young female population. In the present study, we evaluated the predictive value of CRP levels for the risk of ACS and clinical outcomes of long-term follow-up in the Chinese young female population.

## 2. Materials and Methods

### 2.1. Patients

The study was conducted by 1 : 1 case-control to retrospectively analyze 420 young women with ACS (ACS group) who underwent PCI and 420 young women (control group) who underwent coronary angiography (CAG) to exclude CHD by propensity score matching from January 2007 to December 2016. All patients were divided into three subgroups according to risk stratification from the American Heart Association (AHA): subgroup 1 (low risk): CRP < 1.0 mg/L (*n* = 402); subgroup 2 (average risk): 1.0 mg/L ≤ CRP ≤ 3.0 mg/L (*n* = 303); and subgroup 3 (high risk): CRP > 3.0 mg/L (*n* = 135). The levels of serum CRP were observed in the two groups and three subgroups. Baseline characteristics of the enrolled patients were recorded in detail, including age, gender, body mass index (BMI), smoking history, hypertension, diabetes, the level of CRP, and other biomarkers. Exclusion criteria included the following: including congestive heart failure (CHF), myocardiopathy, congenital heart disease, history of aorta surgery, rheumatic heart disease (RHD), severe valve disease, pulmonary or connective tissue disease, active cancer, infective endocarditis, and chronic kidney disease. All the patients were recruited from the Chinese population, and all subjects signed the informed consent. All patients with ACS took prescribed dual antiplatelet therapy, including aspirin (300 mg loading dose, followed by 100 mg once daily) and clopidogrel (300 mg loading dose, followed by 75 mg once daily) for at least 12 months after PCI regardless of drug-eluting stent (DES) type. Individual medical management decisions during hospitalization were exclusively decided by their responsible interventional cardiologists and physicians. Clinical follow-up was performed by telephone contact or outpatient clinical visits. The date of follow-up was ended in December 2018. The primary endpoints for this study were MACE, which included composite occurrence of cardiac death, nonfatal myocardial infarction, and target vessel revascularization (TVR). The follow-up time range was from 11 months to 146 months. The mean duration of follow-up was 66.37 ± 30.06 months.

### 2.2. Statistical Analysis

The sample size of this study was calculated by using Power Analysis and Sample Size software (PASS, v 11.0.10, developed by NCSS, LLC, Kaysville, Utah, USA). The data statistical analyses were performed using the Statistical Package for Social Sciences software (SPSS, version 20.0, SPSS Inc. Chicago, IL, USA). All data of cases were inputted into the computer software database. The continuous variables with normal distributions were expressed as mean ± standard deviation. The comparisons between groups were performed using the independent Student's *t*-test. The counting data were expressed as a percentage (%), and the chi-square (*X*^2^) test was used for comparison between groups. The association of CRP and MACE was investigated by multivariable Cox proportional-hazard models adjusted for all potential confounding factors. The test level was set as a double-tail test *a* = 0.05, *p* < 0.05 was statistically significant, and *p* < 0.01 was statistically very significant.

## 3. Results and Analysis

### 3.1. Baseline Characteristics in ACS Group and Control Group Are Shown in Table 1

There is no significant difference in age between the two groups (*p* > 0.05). Compared with the control group, the proportion of overweight, smoking, hypertension, diabetes, and hypercholesterolemia in the ACS groups is significantly higher (*p* < 0.05). The level of CRP in the ACS group is significantly higher than that in the control group (1.30 ± 1.70 vs. 3.33 ± 5.92, respectively, *p* < 0.001).

### 3.2. Bivariate Logistic Regression Analysis of the Relationship between CRP and ACS Is Shown in Table 2

The result suggests that CRP was an independent predictor of ACS (OR = 1.233, *p* < 0.05).

### 3.3. Baseline Characteristics in Each Subgroup Are Shown in Table 3

There was no significant difference in age, HB, and Hcy in the three subgroups (*p* > 0.05). Overweight, smoking, hypertension, diabetes, hypercholesterolemia, unstable angina, double coronary lesions, three coronary lesions, total stent length, SCr, Uric, LDL, HDL, TC, and TG in subgroup 3 were significantly higher than those in subgroup 1 and subgroup 2 (*p* < 0.05).

### 3.4. Long-Term Clinical Outcomes during Follow-Up Are Shown in Table 4

**Figure 1:**
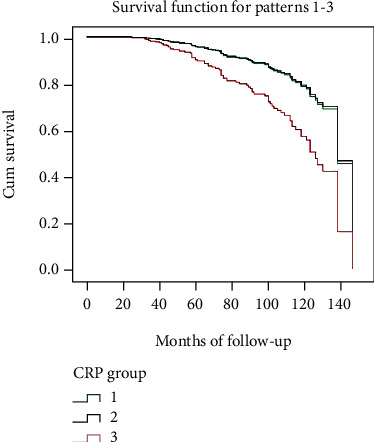
Curve of event-free survival function in three subgroups.

Patients with higher CRP levels (CRP > 3 mg/L) were associated with a significantly increased rate of MACE. Cox regression model analysis illustrated that there were significant increases in MACE in patients with higher CRP during the follow-up (see Figure 1).

## 4. Discussion

CRP is an acute inflammatory response protein induced by cytokines and is secreted by the liver and activated macrophages in atherosclerotic plaques [12]. CRP also plays a direct regulatory role in the process of atherosclerosis, which is related to cytokine release, smooth muscle cell migration, extracellular matrix remodeling, endothelial dysfunction, and the activation of circulating monocytes [13, 14]. Elevated preprocedural CRP is associated with an increased risk for CI-AKI in patients undergoing PCI [15]. CRP is closely related to ACS. CRP promotes the formation of unstable atherosclerotic plaques and triggers the rupture of vulnerable plaques, leading to coronary thrombosis and occurrence of ACS and MACE [16, 17]. Studies have shown that the elevated CRP is closely related to coronary artery events and may be to predict future adverse cardiac events. There was significant difference in serum CRP level between the coronary artery disease (CAD) group and the non-CAD group. The serum CRP level in acute myocardial infarction (AMI), unstable angina, and stable angina pectoris is higher than that in non-CAD patients. It is suggested that CRP is related to the severity of CAD [18]. It is not only an inflammatory product but also a positive feedback to aggravate the inflammatory reaction and atherosclerosis. There is a causal relation between CRP and the pathogenesis of ACS [19–21]. The application of anti-inflammatory drugs (such as colchicine) can reduce the risk of cardiovascular events, which also confirmed that inflammation is involved in the occurrence and development of ACS [22, 23]. Our previous study confirmed that CRP was an independent predictor of young females with ACS [24]. We found that in addition to the traditional risk factors such as overweight, smoking, hypertension, diabetes, and hypercholesterolemia, CRP was also an independent risk factor for ACS in young women. In this further in-depth study, the result showed that the level of CRP in the ACS group was significantly higher than that in the control group (1.30 ± 1.70 vs. 3.33 ± 5.92, respectively, *p* < 0.001). The result of bivariate logistic regression analysis of the relationship between CRP and ACS showed that CRP was an independent predictor suffering from ACS (OR = 1.233, *p* < 0.05). Patients with higher CRP levels (CRP > 3 mg/dL) were associated with a significantly increased rate of MACE which included all-cause death, recurrence of nonfatal myocardial infarction, and target vessel revascularization (TVR) in terms of long-term clinical outcome. Cox regression model analysis illustrate that there was a significant increase rate of MACE in patients with rising CRP during the follow-up. Among the three CRP subgroups, the event-free survival curve was different, and the survival curve of subgroup 3 was significantly lower than that of the other two groups. We believe that CRP, as a useful and easily available biomarker, can be used to improve the risk assessment and secondary prevention of coronary heart disease. At the same time, it was found that there was a good correlation between the CRP level and the severity of coronary artery lesions. CRP level was an independent predictor for the incidence of MACE. Compared with the lower CRP, the patients with higher CRP values had a significantly higher rate of MACE during follow-up [25].

## 5. Conclusions

Our research indicates that high CRP is associated with increased risk of ACS in the Chinese young female population. Risk stratification with CRP as an adjunct to predict clinical risk factors might be useful in the Chinese young female population.

## Figures and Tables

**Table 1:** Baseline characteristics of each group.

	ACS group (420)	Control group (420)	*t*/*x*^2^	*p* value
Age	40.64 ± 4.14	40.77 ± 3.86	0.353	0.742
Overweight	265 (63.10)	70 (16.67)	188.804	≤0.001
Smoking	27 (6.43)	12 (2.86)	6.050	0.014
Hypertension	207 (49.29)	49 (11.67)	140.262	≤0.001
Diabetes	97 (23.10)	17 (4.05)	46.956	≤0.001
Hypercholesterolemia	35 (8.33)	8 (1.90)	17.868	≤0.001
CRP	3.33 ± 5.92	1.30 ± 1.70	6.761	≤0.001

**Table 2:** Bivariate logistic regression analysis of the relationship between CRP and ACS.

	*B*	Wald	*p*	OR	95% CI
CRP	0.209	33.697	≤0.001	1.233	1.15-1.32

**Table 3:** Baseline characteristics of each subgroup.

	Subgroup 1: CRP < 1.0 mg/L	Subgroup 2: 1.0 mg/L ≤ CRP ≤ 3.0 mg/L	Subgroup 3: CRP > 3.0 mg/L	*F*/*X*^2^	*p* value
Number	402	303	135		
Age (years old)	40.83 ± 3.57	40.98 ± 3.99	40.41 ± 4.61	1.010	0.365
Overweight n (%)	134 (33.3)	114 (37.3)	87 (64.4)	41.935	≤0.001
Smoking, *n* (%)	18 (4.48)	14 (4.62)	7 (5.19)	0.118	0.943
Hypertension, *n* (%)	108 (26.87)	84 (27.72)	64 (47.41)	21.846	≤0.001
Diabetes, *n* (%)	44 (10.95)	38 (12.54)	32 (23.70)	14.486	0.001
Hypercholesterolemia, *n* (%)	12 (2.99)	20 (6.60)	11 (8.15)	7.761	0.021
Unstable angina, *n* (%)	154 (38.31)	91 (30.03)	64 (47.41)	12.652	0.002
Double coronary lesions, *n* (%)	24 (5.97)	23 (7.59)	22 (16.30)	14.567	0.001
Three coronary lesions, *n* (%)	5 (1.24)	2 (0.66)	5 (3.70)	6.323	0.042
Total stent length (mm)	10.39 ± 12.06	10.07 ± 12.87	20.16 ± 10.80	41.751	≤0.001
SCr (*μ*mol/L)	61.38 ± 14.63	59.49 ± 11.03	65.26 ± 26.05	6.086	0.002
Uric (*μ*mol/L)	269.56 ± 71.24	289.61 ± 72.77	296.73 ± 88.36	18.119	≤0.001
HB (g/L)	128.14 ± 13.09	130.53 ± 13.67	126.23 ± 15.39	0.147	0.701
Hcy (*μ*mol/L)	9.53 ± 4.87	8.84 ± 3.94	9.91 ± 5.51	0.669	0.414
LDL (mmol/L)	2.40 ± 0.81	2.56 ± 0.83	2.78 ± 1.21	22.904	≤0.001
HDL (mmol/L)	1.19 ± 0.30	1.22 ± 0.35	1.05 ± 10.31	10.028	0.002
TC (mmol/L)	4.10 ± 1.08	4.50 ± 1.12	4.51 ± 1.28	21.039	≤0.001
TG (mmol/L)	1.44 ± 0.87	1.73 ± 1.49	1.78 ± 1.16	13.081	≤0.001

Note: SCr: serum creatinine; HB: hemoglobin; Hcy: homocysteine; Uric: serum uric acid; HDL: high-density lipoprotein; LDL: low-density lipoprotein; TC: total cholesterol; TG: triglycerides.

**Table 4:** Clinical outcomes during follow-up.

	Subgroup 1: CRP < 1.0 mg/L	Subgroup 2: 1.0 mg/L ≤ CRP ≤ 3.0 mg/L	Subgroup 3: CRP > 3.0 mg/L	*X* ^2^	*p* value
Death, *n* (%)	3 (0.75)	1 (0.33)	4 (2.96)	7.210	0.027
MI, *n* (%)	5 (1.24)	0 (0.00)	4 (2.96)	7.931	0.019
TVR, *n* (%)	26 (6.47)	26 (8.58)	22 (16.30)	12.166	0.002
MACE, *n* (%)	28 (6.97)	27 (8.91)	26 (19.26)	17.832	≤0.001

Note: MI: myocardial infarction; TVR: target vessel revascularization; MACE: major adverse cardiac events.

## Data Availability

The data used to support the findings of this study are available from the corresponding authors upon request.
